# When and Why Leaders’ Helping Behavior Promotes Employees’ Thriving: Exploring the Role of Voice Behavior and Perceived Leader’s Role Overload

**DOI:** 10.3389/fpsyg.2020.553512

**Published:** 2020-09-30

**Authors:** Long Chen, Zhen-Duo Zhang, Wen-Tong Jia

**Affiliations:** ^1^Business School, Hohai University, Nanjing, China; ^2^School of Management, Harbin Institute of Technology, Harbin, China; ^3^PE School, Hebei Normal University, Shijiazhuang, China

**Keywords:** perceived leader’s helping behavior, thriving at work, voice behavior, perceived leader’s role overload, social learning theory

## Abstract

Employees who thrive contribute to their organization’s competitive advantage and sustainable performance. The aim of this study was to explore how employees’ thriving is shaped by their leaders’ behavior. Drawing on social learning theory, we examined the relationship between perceived leader’s helping behavior and employees’ thriving. Positing voice behavior as a mediator and perceived leader’s role overload as a moderator, we constructed a moderated mediation model. Using 205 daily data points from 51 employees in various industries, we found that perceived leader’s helping behavior had a positive effect on employees’ thriving at work and that employees’ voice behavior mediated this effect. With the increase of perceived leader’s role overload, the positive relationship between perceived leader’s helping behavior and employees’ voice behavior as well as the indirect effect of perceived leader’s helping behavior on employees’ thriving via employees’ voice behavior were increasingly strong. The findings of our study have implications for research on employees’ thriving at work, leaders’ helping behavior, and social learning theory. There are also practical implications for the behavior of leaders who experience role overload.

## Introduction

In the knowledge-based economy, a thriving workforce is critical for an organization to acquire a competitive advantage and sustainable performance (Spreitzer et al., 2012). Considerable evidence indicates that thriving at work—defined as “the psychological state in which individuals experience both a sense of vitality and a sense of learning at work” (Spreitzer et al., 2005, p. 538)—is associated with numerous positive outcomes, such as higher job satisfaction, less burnout, lower turnover intention, and better task and creative performance (see Kleine et al., 2019, meta-analytic review). Recent studies also indicate that thriving employees are more likely to engage in taking-charge behavior, which can bring constructive change for organizations (Li et al., 2019). Besides the practical benefits, many scholars regard thriving at work as a mediator that can explain the impact of individual characteristics (e.g., Alikaj et al., 2020), leadership (e.g., Niessen et al., 2017; Xu et al., 2017; Li et al., 2019), and organizational contextual factors (e.g., Chang and Busser, 2020; Jiang et al., 2020) on favorable organizational outcomes (e.g., creativity, job satisfaction, and taking charge). For example, Hildenbrand et al.’s (2018) empirical study demonstrated that transformational leadership can decrease burnout via enhancing thriving at work. Therefore, identifying the contributing factors of thriving at work has both practical and theoretical implications.

For this reason, extensive research has identified several predictors of employees’ thriving at work, which can be categorized into three broad aspects (see Table 1). The first aspect focuses on individual factors, which include individual characteristics (e.g., Paterson et al., 2014; Alikaj et al., 2020), emotions (e.g., Porath et al., 2012), perceptions (e.g., Jiang et al., 2019; Kim and Beehr, 2020), attitudes (e.g., Van der Walt, 2018), and behavior (e.g., Niessen et al., 2012; Sun et al., 2019). For example, scholars found that proactive personality (Alikaj et al., 2020), positive affect (Porath et al., 2012), psychological safety (Jiang et al., 2019), job engagement (Van der Walt, 2018), and agentic work behavior (Niessen et al., 2012) have a positive impact on employees’ thriving. The second aspect attempts to construct the impact of leaders’ behavior and characteristics on employees’ thriving at work (e.g., Li et al., 2016; Niessen et al., 2017; Walumbwa et al., 2018). For example, it has been shown that authentic leadership (Xu et al., 2017), empowering leadership (Li et al., 2016), service leadership (Jo et al., 2020), and transformational leadership (Lin et al., 2020) are all positively related to employees’ thriving. The third part of the research explores the effect of job and organizational factors (e.g., Prem et al., 2017; Jiang et al., 2020; Strecker et al., 2020). Along with this research stream, some scholars demonstrated that job autonomy (Strecker et al., 2020), workplace civility (Abid et al., 2018), and organizational justice (Kim and Beehr, 2020) are key variables in fostering employees’ thriving.

**TABLE 1:** Antecedent variables of thriving at work in previous research.

**Categories**	**Antecedent variables**	**Examples of empirical research**
Individual factors	Proactive personality	Alikaj et al., 2020
	Psychological capital	Paterson et al., 2014
	Core self-evaluations	Zhu et al., 2018
	Positive affect and negative affect	Porath et al., 2012
	Psychological safety	Jiang et al., 2019
	Psychological empowerment	Kim and Beehr, 2020
	Job engagement	Van der Walt, 2018
	Agentic work behavior	Niessen et al., 2012
	International business traveler frequency	Dimitrova, 2020
	Work-related usage of social media	Sun et al., 2019
	Helping neighbors	Zhang et al., 2020
Leader’s factors	Authentic leadership	Xu et al., 2017
	Empowering leadership	Li et al., 2016
	Ethical leadership	Yousaf et al., 2019
	Leader inclusiveness	Li et al., 2019
	Leader–member exchange	Xu et al., 2019
	Managerial coaching	Raza et al., 2018
	Paradoxical leader behavior	Yang et al., 2019
	Service leadership	Jo et al., 2020
	Supervisor prosocial motivation	Frazier and Tupper, 2018
	Supervisor support	Russo et al., 2018
	Transformational leadership	Lin et al., 2020
Job and organizational factors	Task identity	Jiang et al., 2020
	Autonomy	Strecker et al., 2020
	Job stress	Prem et al., 2017
	Organizational justice	Kim and Beehr, 2020
	Social and organizational support	Chang and Busser, 2020; Strecker et al., 2020
	Support climate	Paterson et al., 2014
	Workplace civility	Abid et al., 2018
	Psychological contract fulfillment	Chang and Busser, 2020
	Trust	Carmeli and Spreitzer, 2009

*Sorting and updating was done on the basis of Kleine et al. (2019) meta-analysis.*

Despite the abundant findings on the predictors of thriving at work, further research needs to examine other important antecedents, such as perceived leader’s helping behavior, which is defined as the extent to which employees perceive their leaders as voluntarily assisting them in work-related areas (Anderson and Williams, 1996). Helping behavior is a crucial constituent of a leadership role (Nadler et al., 2003; Drach-Zahavy and Somech, 2006), and leaders should allocate appropriate time to help their subordinates in tackling task and personal issues (Anderson, 1992). As such, leaders’ helping behavior is a pervasive phenomenon in the workplace, and there is a body of literature that has documented its benefits (e.g., Price and Vugt, 2014; Asadullah et al., 2016; Hoption, 2016). For example, Asadullah et al. (2016) found that leaders’ helping behavior was associated with leader effectiveness, and Hoption (2016) suggested that when receiving more help from their leaders, employees were more likely to appraise their relationship with leaders as satisfactory. Considering that helping behavior is indispensable in acting in a leadership role (Anderson, 1992; Nadler et al., 2003; Drach-Zahavy and Somech, 2006), it is valuable to understand the impact of perceived leaders’ helping behavior. In this study, we aimed to reveal the function of perceived leaders’ helping behavior in cultivating employees’ thriving at work, which has been overlooked in previous research.

In addition to linking perceived leaders’ helping behavior and employees’ thriving at work, this study further took its mechanism and boundary condition into account by drawing on the perspective of social learning theory, which argues that people are most likely to learn the behavior that can produce valued outcomes via role modeling (Bandura, 1977; Wood and Bandura, 1989). In the workplace, leaders have the power to control rewards (Bandura, 1986; Brown et al., 2005). Therefore, modeling behavior on leaders is important for employees to acquire rewards and avoid punishment, and leaders generally serve as employees’ role models (Brown et al., 2005). Based on social learning theory, we propose that when leaders offer assistance in achieving employees’ tasks, employees tend to model the leader’s affiliative helping behavior and engage in affiliative behavior targeted at helping to improve organizational functions. Employees will experience a sense of learning and competence (Wood and Bandura, 1989), which can foster thriving at work (Spreitzer et al., 2005; Cangiano et al., 2019). Hence, employees’ affiliative behavior as a result of learning from leaders can illustrate the effect of perceived leader’s helping behavior on thriving at work. Considering that people want to obtain instructive feedback during the process of learning (Bandura, 1977), we argue that the affiliative behavior modeled by employees should make it easier to acquire leaders’ feedback. Voice behavior, which is defined as employees’ communication behavior of expressing challenging but constructive suggestions intended to improve current organizations rather than merely criticize, is a kind of affiliative promotive behavior (Van Dyne and LePine, 1998). Moreover, as employees’ ideas are generally reported to their leaders (Detert and Burris, 2007), voice behavior is more likely to help employees obtain instructive feedback. Thus, we assumed that voice behavior is appropriate to act as a mediator associated with the relationship between perceived leader’s helping behavior and thriving at work.

Furthermore, this study investigated perceived leader’s role overload, which refers to employees’ perception that their leader is expected to fulfill too many responsibilities or activities (Rizzo et al., 1970), as a boundary condition for the relationship between perceived leader’s helping behavior and employees’ voice behavior. Since social learning theory posits that people have a high tendency to learn behavior that is valuable (Bandura, 1977), this study assumed that employees are more likely to model behavior that is encouraged and emphasized by leaders. Besides that, it is a universal logic that leaders will prioritize actions that they deem valuable and important when there is too much work to be done, i.e., role overload (Becker, 1965). Corresponding with the above logic, employees will evaluate leaders’ behavior as worthy and have a high probability of modeling leaders’ behavior when perceiving that their leaders are experiencing role overload. Therefore, the impact of perceived leader’s helping behavior on employees’ voice behavior, as well as the indirect effect of perceived leader’s helping behavior via voice behavior on thriving at work, might be stronger among employees who perceive that their leaders have a high role overload.

**FIGURE 1:**
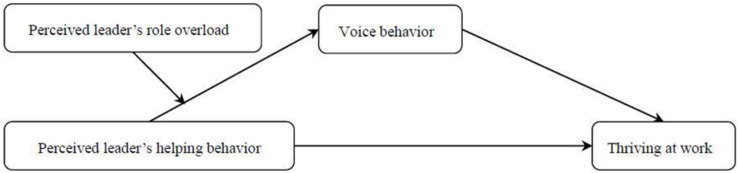
Theoretical model.

This study constructed a moderated mediating model as shown in Figure 1. Different from previous studies on leaders’ behavior and employees’ thriving, we focus on how the employee’s daily thriving is shaped by the leader’s daily helping behavior. To examine our model, we measured perceived leader’s helping behavior and perceived leader’s role overload every morning and employees’ voice behavior and thriving at work every afternoon over five consecutive workdays. This research design has an advantage in observing the relationship between within-individual variables.

By designing the moderated mediating model, this study contributes to previous literature in three ways. First, this study tried to extend the antecedents of thriving by adding perceived leader’s helping behavior. Previous research has shown that leadership is one of the key aspects influencing thriving at work (e.g., Li et al., 2016; Xu et al., 2017; Jo et al., 2020). Our research is one of the first studies to bridge the link between perceived leader’s helping behavior and thriving at work. By doing this, we can also highlight the benefits of leaders’ helping behavior in fostering employees’ thriving. Second, this study offers a new perspective to explain the impact of leaders’ behavior on thriving at work drawing from social learning theory. Prior scholars have identified the socially embedded model and self-determination theory as the theoretical perspective that can demonstrate how thriving at work is influenced by leaders’ behavior (e.g., Xu et al., 2017, 2019; Li et al., 2019). The findings of this study could emphasize the mediating role of voice behavior and advance our understanding of the process by which perceived leader’s helping behavior affects thriving at work. Third, this study examined the boundary condition when perceived leader’s helping behavior enhances employees’ thriving via voice behavior by accounting for the moderating role of perceived leader’s role overload. Further, this finding might contribute to social learning theory by considering leaders’ role overload as a clue when employees imitate the behavior of their leaders.

## Theory and Hypotheses

### Perceived Leader’s Helping Behavior and Thriving at Work

Thriving at work is a joint experience of learning and vigor (Spreitzer et al., 2005). Employees who are thriving should experience both a feeling of vigor and a feeling of learning at work (Spreitzer et al., 2005). Thriving is a positive and activated psychological state, such that vigor refers to the sense of liveliness and activeness (Ryan and Frederick, 1997; Nix et al., 1999) and learning refers to the cognitive belief in terms of acquiring and using knowledge and skills (Carver, 1998; Spreitzer et al., 2005). According to the argument that thriving employees should experience progress as well as energy (Spreitzer et al., 2012), neither employees who maintain a state of learning but feel exhausted nor employees who have vitality to work but lack the opportunity to learn and promote personal growth can be regarded as thriving.

It is increasingly being discussed how thriving at work is influenced by leaders’ behavior (Li et al., 2016; Niessen et al., 2017; Walumbwa et al., 2018). However, no research addresses the impact of leaders’ helping behavior, which is a key ingredient of a leadership role (Nadler et al., 2003; Drach-Zahavy and Somech, 2006). Leaders’ helping behavior is described as the leader assisting subordinates in tasks (Anderson and Williams, 1996). For leaders, it is a kind of prosocial and affiliative behavior that is not required in the formal performance evaluation (Van Dyne and LePine, 1998). Generally speaking, helping behavior, which includes acts of consideration, is promotive and cooperative in nature (Van Dyne and LePine, 1998). Previous research indicated that leaders’ helping behavior did benefit in promoting leader effectiveness and relationship satisfaction (Asadullah et al., 2016; Hoption, 2016). In line with these studies, we argue that perceived leader’s helping behavior has a positive effect on employees’ thriving and can be explained from the following two perspectives.

First, leaders’ helping behavior can act as a workplace resource that enables employees to engage in agentic work behaviors. The core content of leaders’ helping behavior is to assist employees in completing tasks (Anderson and Williams, 1996). With a leader’s help, employees will obtain more information and better know how tasks should be carried out, so that their knowledge resources are increased (Orlikowski, 2002). Besides that, leaders’ helping behavior contributes to building a high-quality relationship between the leader and the employee who receives help (Van Dyne and LePine, 1998). As indicated in Spreitzer et al.’s (2005) study, the relationship between leader and subordinate can be regarded as a relational resource for the subordinate. According to the socially embedded model of thriving at work (Spreitzer et al., 2005), both knowledge and relational resources can encourage employees to engage in active and purposeful work behavior, i.e., agentic work behavior (Bandura, 2001). Further, numerous empirical studies have offered support for the positive role of these agentic work behaviors in employees’ thriving (e.g., Niessen et al., 2012; Paterson et al., 2014). Therefore, there should be a positive link between perceived leader’s helping behavior and employees’ thriving at work from the perspective of the socially embedded model.

Second, self-determination theory suggests that the leader plays an important role in satisfying the employee’s basic psychological needs (Deci and Ryan, 2000), and we assume that the leader’s helping behavior might satisfy the employee’s need for autonomy, competence, and relatedness. As mentioned above, leaders’ helping behavior offers knowledge resources for employees. Using these knowledge resources, the employee knows the best practice of completing work successfully. As such, the employee can complete work in a shorter time and allocate spare time to doing something autonomously. When work is completed, the employee will feel a sense of accomplishment (Spreitzer et al., 2005), and the need for feeling competent is satisfied. Finally, the employee’s need for relatedness can also be satisfied by the leader’s helping behavior because helping behavior enables helpers and recipients to build high-quality relationships (Van Dyne and LePine, 1998). Evidence has shown that employees will experience vigor and learning when their basic needs are satisfied (Ryan et al., 2010; Janke et al., 2015). Hence, we proposed the following hypothesis:

Hypothesis 1: Perceived leader’s helping behavior is positively associated with employees’ thriving.

### The Mediating Role of Voice Behavior

In addition to the above two perspectives, we can also illustrate the positive link between perceived leader’s helping behavior and employees’ thriving from the perspective of social learning theory. Social learning theory argues that people can learn new behavior via observation and that the behavior that people imitate should produce valuable and rewarded outcomes (Bandura, 1977). People generally model their behavior on a person who has power and high standing because of his/her ability to control the rewards (Bandura, 1986). Besides the behavior, people also learn the rules behind the modeled behavior, and they can use the rules to generate new behavior (Wood and Bandura, 1989). For example, when observing a leader’s helping behavior, employees might not only imitate helping behavior but also learn the affiliative rule that guides employees to conduct other kinds of affiliative behavior (e.g., voice behavior). As leaders are more powerful and able to control the rules of reward and punishment (Brown et al., 2005), employees are more likely to learn from leaders. When leaders help employees to complete tasks, employees will learn the rules that affiliative behavior, which contributes to organizational effectiveness, is encouraged and valued by leaders. Then, these employees tend to engage in affiliative behavior. The theory also supports the view that observing prosocial behavior motivates people to learn such behavior (Bryan and Test, 1967). Due to the fact that imitation is a process of learning and helps people to acquire competence (Wood and Bandura, 1989), engaging in affiliative behavior as a way of learning a leader’s helping behavior makes employees feel a sense of growth and competence. Evidence also confirmed that competence enables employees to feel validity (Cangiano et al., 2019). Hence, perceived leader’s helping behavior can increase thriving at work through encouraging affiliative behavior, which is modeled on that of the leader.

Nevertheless, it is inappropriate to assume that all of the affiliative behavior can be carried out in the process of learning from leaders’ helping behavior. In order to confirm the effectiveness of learning and imitation, people need instructive feedback after practicing new rules or skills (Wood and Bandura, 1989). As such, employees should engage in the affiliative behavior that can draw leaders’ attention. Voice behavior is a kind of affiliative behavior, which is defined as “promotive behavior that emphasizes expression of constructive challenge intended to improve rather than merely criticize” (Van Dyne and LePine, 1998, p. 109). Voice behavior generally involves the ideas of improving current organizational policies and practices that are formed by leaders (Detert and Edmondson, 2011; Liang et al., 2012). Leaders should evaluate the new ideas and might give some feedback when employees voice change-oriented ideas. Hence, leaders often serve as the targets of voice (Detert and Burris, 2007), and employees who speak up frequently have more opportunities to be noticed as well as acquire leaders’ feedback. The above arguments lead us to conclude that employees have a high probability of modeling leaders’ helping behavior by voice behavior and then experiencing thriving at work.

In the following part, we will explain how voice behavior mediates the relationship between perceived leader’s helping behavior and employees’ thriving according to social learning theory. Perceiving that leaders frequently perform helping behavior, employees will learn the rules that leaders are following in terms of the affiliative prosocial behavior that can promote organizational effectiveness. Guided by these rules, employees will actively generate useful ideas and speak out to help achieve organizational goals. Accordingly, voice behavior during the process of learning from leaders is a kind of proactive behavior, which means that employees have the competence to change and control the current environment (Cangiano et al., 2019). Meanwhile, employees who express new ideas will definitely approach new ways and procedures for work. Previous studies have documented that both feeling competence and being exposed to novelty enable employees to experience validity (Kaplan and Kaplan, 1995; Cangiano et al., 2019). Besides that, voice behavior is a process to share employees’ thoughts about organizational development, and it is necessary for them to develop new ideas and novel strategies (Farh et al., 2010). It is obvious that these new ideas and novel strategies increase the knowledge that employees can use. As a result of learning a leader’s helping behavior, voice behavior can help employees experience a sense of both validity and learning new things. Therefore, we formulated the following hypothesis:

Hypothesis 2: Voice behavior mediates the positive relationship between perceived leader’s helping behavior and employees’ thriving.

### The Moderating Role of Perceived Leader’s Role Overload

Social learning theory argues that individuals do not learn every behavior they have observed and that only the behavior that produces rewarded and valued outcomes can be learned (Bandura, 1977). Along with social learning theory, we believe that employees learning from leaders’ helping behavior has a weak effect and that the positive impact of perceived leader’s helping behavior on employees’ voice behavior is also small. On the one hand, assisting subordinates to complete tasks is a required behavior for a leader (Nadler et al., 2003; Drach-Zahavy and Somech, 2006). Hence, employees might think that leaders just fulfill their duty when they offer assistance in a work-related area. That is to say, employees may regard leaders’ helping behavior as a regular and normal activity, which is hardly modeled by employees. On the other hand, voice behavior is a challenging behavior and may be interpreted as bossiness by leaders (Detert and Edmondson, 2011). As such, employees would speak out their ideas only when they have clear clues that indicate that the affiliative helping behavior is encouraged by leaders.

The theoretically small effect of leaders’ helping behavior on employees’ voice behavior urges us to find a moderator that can add prominence to the value of learning the leader’s helping behavior. Considering that people usually prioritize the most valuable actions when they are faced with many demands on their time (Becker, 1965) and that a leader’s role overload illustrates a scenario where the leader has to deal with too many responsibilities and activities (Rizzo et al., 1970), we suggest that the leader’s behavior under the condition of role overload is valued and encouraged by leaders. Therefore, employees are more likely to imitate leaders’ behavior when employees know that leaders have too many role demands. Under the scenario of perceived high leader’s role overload, a leader’s helping behavior sends out a clear signal to employees that the leader attaches high value to altruistic and affinitive behavior. Driven by this signal, employees are willing to learn and master the affiliative rules valued by leaders and then have a high tendency to engage in affinitive voice behavior. For example, leaders want to promote team performance, even though they experience role overload. In some cases, leaders may choose to help their subordinates with the aim of avoiding team performance reduction. The subordinates who receive leaders’ help tend to realize that leaders who experience role overload preferentially pay attention to team performance, and thereby, behavior associated with increasing performance is inspired. In order to create an impression of “good soldiers” for leaders, employees will imitate their leaders and share more constructive ideas that can improve team performance. Thus, the association between perceived leader’s behavior and employees’ voice behavior is stronger when the perceived leader’s role overload is at high levels, and we therefore formed the following hypothesis:

Hypothesis 3: Perceived leader’s role overload strengthens the positive relationship between perceived leader’s helping behavior and voice behavior.

Based on all the above mentioned arguments, we proposed a moderated mediating model. Specifically, when perceiving high leader’s role overload, employees will acknowledge clear clues that affiliative behavior is valued by their leaders and will be more willing to master the affiliative rules underlying the helping behavior. Hence, perceived leader’s helping behavior have a larger impact on subsequent voice behavior. By conducting more voice behavior, employees will get access to more new knowledge, ideas, and strategies. This enables employees to experience a better sense of learning. Meanwhile, employees will perceive higher competence and keep more energy via conducting more challenge-oriented voice behavior (Deci and Ryan, 2000). Further, employees should perceive a better sense of learning and mastering due to our theoretical logic that voice behavior is a process of learning from leaders. Hence, the mediating role of voice behavior can be amplified by perceived leader’s role overload, and we formed the following hypothesis:

Hypothesis 4: Perceived leader’s role overload strengthens the positive relationship between perceived leader’s helping behavior and employees’ thriving.

## Materials and Methods

### Participants and Procedure

We gained access to potential participants via personal relationships. Specifically, we contacted the authors’ former classmates who were working in enterprises and asked these classmates to invite their colleagues to participate in this 5-day survey. We gathered the authors’ former classmates and their colleagues who agreed to participate in the study into a WeChat group. These participants were from various industries in China such as manufacturing, education, pharmaceutics, finance, and accounting. Almost all of the participants were staff who did not occupy a management position. We designed three questionnaires, which were each published on a websites. The website with each questionnaire was sent to participants via the WeChat group, and participants could record their responses by visiting these websites. The first questionnaire was used to record participants’ demographic information (i.e., gender, age, education, and organizational tenure). We sent out this questionnaire on the morning of the first workday. The second questionnaire included scales of perceived leader’s helping behavior and perceived leader’s role overload, and we sent out this questionnaire every morning for five consecutive workdays. The third questionnaire included scales of felt obligation, voice behavior, and thriving at work, and we sent out this questionnaire every evening for five consecutive workdays. We will offer a detailed explanation in the following section about why we included scales of felt obligation. In order to match up the different questionnaires answered by each participant, we asked them to record the last two digits of their mobile numbers, birth year, and birth month in each questionnaire.

In total, 110 participants recorded partial or full responses. We included participants who completed questionnaires for 3 days and above, as three data points and above per participant allows for the appropriate modeling of within-person relationships and for capturing the real experiences of working professionals (Bolger and Laurenceau, 2013). Finally, we obtained 205 daily data points from 51 participants. Among these participants, 51.0% were female, and most had a bachelor’s degree (62.7%; 25.5% had a master’s degree; 11.8% had a college degree or lower). The average age was 29.160 years (*SD* = 4.688), and the average organizational tenure was 4.243 years (*SD* = 5.356).

### Measurement

All the scales in this study are originally in English. In order to keep the accuracy of translation, we adopted Brislin’s (1980) principle of translation and back-translation. Specifically, one bilingual professor first translated the English scales into Chinese. Then, another professor and two Ph.D. students (all bilingual) translated these Chinese scales back into English. Finally, these four translators compared the translated scales and the original scales. For any differences, they discussed and made some revisions.

#### Perceived Leader’s Helping Behavior

Three items from Ragins et al.’s (2017) study were adapted. We chose this scale because it has a high reliability and can be used to capture the behavior exhibited by participants’ leaders (Ragins et al., 2017). As the original scale was developed to measure holding behavior, we selected three items that can directly reflect the leader’s assisting in work. Further, we modified the scale so that all items could capture participants’ daily perception of the leader’s helping behavior. The items were “Today, my leader enables me and gives me new perspectives on disturbing or confusing things that happen at work,” “Today, my leader helps me make sense of confusing or upsetting things that happen at work,” and “Today, my leader offers me support when I am faced with upsetting or stressful workplace experiences.” Participants were asked to rate the extent to which they agree with these views. A five-point Likert scale was used to record participants’ responses, with “1” representing “strongly disagree” and “5” representing “strongly agree.” This three-item scale for perceived leader’s helping behavior yielded a Cronbach’s alpha reliability coefficient of 0.898.

#### Thriving at Work

We selected Porath et al.’s (2012) scale to measure the overall construct of thriving at work because this scale was widely used in previous studies and has a high reliability (e.g., Hildenbrand et al., 2018; Li et al., 2019). However, Porath et al.’s (2012) original scale contains 10 items, which might cause participants to experience fatigue, especially in diary research (Ohly et al., 2010). It is recommended that the scales used in diary research should use fewer items, which can reduce participants’ survey fatigue (Ohly et al., 2010; Cangiano et al., 2019). Therefore, we chose the four items that had the highest factor load in the initial scale. We also modified the scale so that all items could capture participants’ daily thriving experience. Two items were used to measure the dimension of learning. The items were “Today, I continue to learn more as time goes by” and “Today, I see myself continually improving.” Two items were used to measure the dimension of vigor. The items were “Today, I feel alive and vital” and “Today, I have energy and spirit.” Participants were asked to rate the extent to which they agree with these views. A five-point Likert scale was used to record participants’ responses, with “1” representing “strongly disagree” and “5” representing “strongly agree.” This four-item scale for thriving at work scale yielded a Cronbach’s alpha reliability coefficient of 0.846.

#### Voice Behavior

We selected Van Dyne and LePine’s (1998) scale to measure voice behavior because this scale was widely used in previous research and has a high reliability (e.g., Venkataramani et al., 2016; Ng et al., 2019). Similarly, we only chose two items from the six-item scale because the length may increase participants’ fatigue in diary research (Ohly et al., 2010; Cangiano et al., 2019). Both the items had the highest factor load in the initial scale. We modified the scale so that the items could capture participants’ daily voice behavior. The items were “Today, I speak up in this organization with ideas for new projects or changes in procedures” and “Today, I develop and make recommendations concerning issues that affect this organization.” Participants were asked to rate how often they engaged in these behaviors today. A five-point Likert scale was used to record participants’ responses, with “1” representing “never” and “5” representing “extremely often.” This two-item voice behavior scale yielded a Cronbach’s alpha reliability coefficient of 0.886.

#### Perceived Leader’s Role Overload

We used Bolino and Turnley’s (2005) three-item scale to measure perceived leader’s role overload because this scale is economic and has a high reliability (Bolino and Turnley, 2005). We modified the scale so that all items could capture participants’ daily perception on leaders’ role overload. The items were “Today, the amount of work my leader expected to do is too great,” “Today, I feel that my leader does not have enough time to get everything done at work,” and “Today, it often seems like my leader has too much work to do.” Participants were asked to rate the extent to which they agree with these views. A five-point Likert scale was used to record participants’ responses, with “1” representing “strongly disagree” and “5” representing “strongly agree.” This three-item scale for perceived leader’s role overload yielded a Cronbach’s alpha reliability coefficient of 0.747.

#### Control Variables

The demographic variables such as gender, education, and organizational tenure were selected as control variables, which were also controlled in other research on thriving at work. In addition, voice behavior is the mediator in this study, and it is necessary to control the factors that can influence voice behavior. As voice behavior is a kind of affiliative pro-organizational behavior, voice behavior can be motivated by the belief that employees have an obligation to do things that benefit the organization. In order to rule out this path, we controlled for felt obligation. As Eisenberger et al.’s (2001) scale has a high reliability and is widely used in prior literature (e.g., Liang et al., 2012; Basi, 2017), we selected three items from the seven-item scale as the measurement of felt obligation. By doing this, we reduced the survey length, as long surveys may increase participants’ fatigue in diary research (Ohly et al., 2010; Cangiano et al., 2019). All three items had the highest factor load in the initial scale. We also modified the scale so that all items could capture participants’ daily experience of obligation. The items were “Today, I feel a personal obligation to do whatever I can to help the organization to achieve its goal,” “Today, I have an obligation to the organization to ensure that I produce high-quality work,” and “Today, I owe it to the organization to do what I can to ensure that customers are well-served and satisfied.” Participants were asked to rate the extent to which they agree with these views. A five-point Likert scale was used to record participants’ responses, with “1” representing “strongly disagree” and “5” representing “strongly agree.” This three-item scale for felt obligation yielded a Cronbach’s alpha reliability coefficient of 0.847.

## Results

As our data are nested, we used hierarchical linear modeling to address this problem (Raudenbush and Bryk, 2002). Perceived leader’s helping behavior, voice behavior, perceived leader’s role overload, and felt obligation were included in Level 1. Demographic factors, including gender, education, and organizational tenure, were Level 2 variables. We first computed the proportion of within-individual variance in all Level 1 variables so that we could understand whether our data are suitable for running hierarchical linear modeling. As shown in Table 2, the percentage of within-individual variance ranges from 48.4 to 50.5%, which indicates that there are advantages to adopting hierarchical linear modeling in analyzing our data. The means, standard deviations, zero-order correlations, and Cronbach’s alphas for within- and between-person levels are shown in Tables 3, 4.

**TABLE 2:** Parameter estimates and variance partitioning of null models for Level 1 variables.

**Variable**	**Intercept**	**Within-individual variance**	**Between-individual variance**	**Percentage of within-individual variance**
Perceived leader’s helping behavior	2.632***	0.626	0.635	49.6%
Thriving at work	3.228***	0.459	0.485	48.6%
Voice behavior	2.868***	0.657	0.645	50.5%
Perceived leader’s role overload	2.995***	0.388	0.398	49.4%
Felt obligation	3.843***	0.297	0.317	48.4%

Level 1 n = 205, ***p < 0. 001.

**TABLE 3:** Within-individual descriptive statistics and correlations.

**Variables**	**1**	**2**	**3**	**4**	**5**
1. Perceived leader’s helping behavior	(0.898)				
2. Thriving at work	0.288***	(0.846)			
3. Voice behavior	0.071	0.140*	(0.886)		
4. Perceived leader’s role overload	0.277***	–0.081	0.110	(0.747)	
5. Felt obligation	0.132	–0.119	0.101	–0.020	(0.847)
Mean	2.637	3.236	2.878	2.997	3.850
S.D.	0.901	0.790	0.901	0.716	0.638

N = 205, *p < 0.05, ***p < 0.001. The values in the parentheses represent Cronbach’α reliability coefficient. All above variables are within-individual variables. The variables were centered at the person level before the within-person correlations were computed. The means and standard deviations are based on between-person scores.

**TABLE 4:** Between-individual descriptive statistics and correlations.

**Variables**	**1**	**2**	**3**	**4**	**5**	**6**	**7**	**8**	**9**
1. Perceived leader’s helping behavior	(0.898)								
2. Thriving at work	0.267	(0.846)							
3. Voice behavior	0.667***	0.510***	(0.886)						
4. Perceived leader’s role overload	0.559***	0.284*	0.404**	(0.747)					
5. Felt obligation	0.267	0.683***	0.476***	0.287*	(0.847)				
6. ^*a*^Gender	0.271	0.132	0.263	–0.072	0.125	−			
7. Age in years	–0.062	–0.174	–0.094	0.096	–0.073	–0.009	−		
8. ^*b*^Education	–0.006	0.443**	0.046	0.288*	0.456**	0.028	–0.029	−	
9. Organizational tenure in years	–0.006	–0.164	0.040	–0.005	–0.119	–0.064	0.855***	–0.261	−
Mean	2.637	3.236	2.878	2.997	3.850	−	29.160	−	4.243
S.D.	0.901	0.790	0.901	0.716	0.638	−	4.688	−	5.356

N = 51, *p < 0.05, **p < 0.01, ***p < 0.001. The values in the parentheses represent Cronbach’α reliability coefficient. ^*a*^Gender (“0,” male; “1,” female). ^*b*^Education (“1,” college and below; “2,” bachelor’s; “3,” master’s and above). Variable 1, variable 2, and variable 3 are within-individual variables. Variables 4 through 8 are between-individual variables. Correlations are based on between-individual scores. To achieve this, all Level 1 variables (variables 1 through 3) were aggregated to the individual level.

Considering the nested nature of our data, we conducted a two-level confirmatory factor analysis (CFA) to examine the construct validity for perceived leader’s helping behavior, thriving at work, voice behavior, perceived leader’s role overload, and felt obligation. As we only focused on the broad construct of thriving at work, we computed the scale score of learning and vigor and loaded these on thriving at work. The results of the two-level CFA are shown in Table 5. Among all the measurement models, the hypothesized five-factor measurement model (containing perceived leader’s helping behavior, thriving at work, voice behavior, perceived leader’s role overload, and felt obligation) fits the data best (χ^2^ = 180.783, *df* = 110, *CFI* = 0.933, *TLI* = 0.905, *RMSEA* = 0.056). The results of the CFA showed that the five variables above have construct validity.

**TABLE 5:** Results of multilevel confirmative factor analysis.

**Model**	**χ ^2^**	**df**	**CFI**	**TLI**	**RMSEA**	**△χ ^2^(df)**
One-factor model	674.039	130	0.483	0.380	0.143	–
Two-factor model	505.216	128	0.642	0.563	0.120	168.823 (2)***
Three-factor model	499.368	124	0.643	0.551	0.122	5.848 (4)
Four-factor model	276.024	118	0.850	0.801	0.081	223.344 (4)***
Five-factor model	180.783	110	0.933	0.905	0.056	95.241 (8)***

One-factor model: perceived leader’s helping behavior + thriving at work + voice behavior + perceived leader’s role overload + felt obligation. Two-factor model: perceived leader’s helping behavior + perceived leader’s role overload, thriving at work + voice behavior + felt obligation. Three-factor model: perceived leader’s helping behavior + perceived leader’s role overload, thriving at work + voice behavior, felt obligation. Four-factor model: perceived leader’s helping behavior + perceived leader’s role overload, thriving at work, voice behavior, felt obligation. Five-factor model: perceived leader’s helping behavior, thriving at work, voice behavior, perceived leader’s role overload, felt obligation. ***p < 0.001.

The results of the hierarchical linear model are shown in Table 6. Model 3 in Table 6 shows that perceived leader’s helping behavior was significantly associated with thriving at work (*B* = 0.239, *SE* = 0.057, *p* < 0.001). Hypothesis 1 was therefore supported. Model 4 shows that perceived leader’s helping behavior was significantly associated with voice behavior (*B* = 0.250, *SE* = 0.088, *p* < 0.01). Model 5 shows that voice behavior was significantly associated with thriving at work (*B* = 0.161, *SE* = 0.061, *p* < 0.01). Using R software, we conducted a parametric bootstrapping and computed the indirect effect of perceived leader’s helping behavior on thriving at work via voice behavior. Results showed that this indirect effect is significantly different from zero (Indirect = 0.040, *SE* = 0.022, 95% confidence interval [CI] for the indirect effect [0.001, 0.092]). Hence, Hypothesis 2 was supported.

**TABLE 6:** Results of hierarchical linear model.

**Variables**	**Model 1**	**Model 2**	**Model 3**	**Model 4**	**Model 5**	**Model 6**	**Model 6**
	
	**Thriving at work**			**Voice behavior**	**Thriving at work**	**Voice behavior**	**Thriving at work**
Intercept	3.228 (0.109)***	1.678 (0.565)**	1.311 (0.458)**	0.940 (0.448)*	1.099 (0.547)*	0.534 (0.532)	1.393 (0.619)*
**Level 2 predictors**							
^*a*^Gender		0.165 (0.182)	0.057 (0.183)	0.336 (0.184)	−0.001 (0.189)	0.399 (0.188)*	−0.050 (0.179)
^*b*^Education		0.527 (0.153)**	0.570 (0.163)**	−0.034 (0.213)	0.568 (0.142)**	−0.124 (0.209)	0.639 (0.156)***
Organizational tenure in years		−0.006 (0.015)	−0.006 (0.018)	0.013 (0.010)	−0.008 (0.012)	0.009 (0.011)	−0.006 (0.013)
**Level 1 predictors**							
Felt obligation		0.095 (0.133)	0.017 (0.084)	0.288 (0.112)*	−0.012 (0.128)	0.277 (0.103)**	−0.027 (0.132)
Perceived leader’s helping behavior			0.239 (0.057)***	0.250 (0.088)**	0.202 (0.061)**	0.178 (0.089)*	0.245 (0.063)***
Voice behavior					0.161 (0.061)**		0.186 (0.055)**
Perceived leader’s role overload						0.251 (0.095)**	−0.177 (0.096)
Perceived leader’s helping behavior × perceived leader’s role overload						0.158 (0.073)*	−0.051 (0.065)
σ^2^	0.458	0.472	0.425	0.680	0.418	0.638	0.402
τ (intercept)	0.485	0.302	0.309	0.288	0.271	0.291	0.280
N (Level 1)	205	205	205	205	205	205	205
N (Level 2)	51	51	51	51	51	51	51
−2 log likelihood	504.149	490.405	473.755	551.208	465.949	540.534	460.660

*p < 0.05, **p < 0.01, ***p < 0. 001. All data are unstandardized estimates, and the values in parentheses represent the standard error of the unstandardized regression coefficient. ^*a*^Gender (“0,” male; “1”, female). ^*b*^Education (“1,” college and below; “2,” bachelor’s; “3,” master’s and above).

**FIGURE 2:**
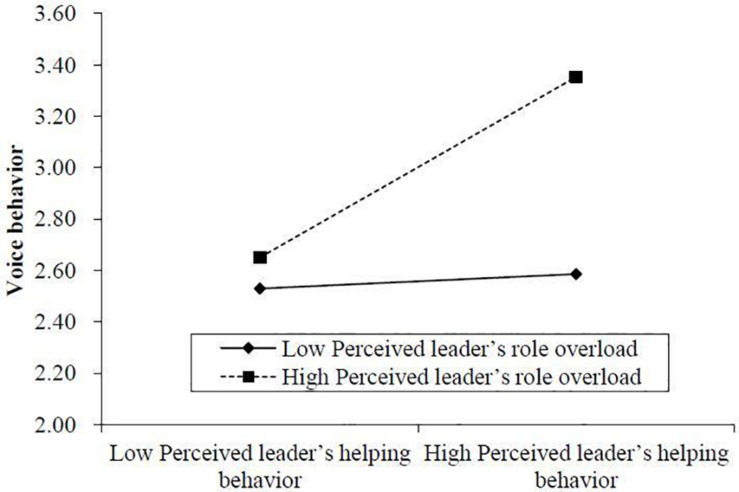
The moderating effect of perceived leader’s role overload on the relationship between perceived leader’s helping behavior and voice behavior.

To examine the moderating effect of perceived leader’s role overload, we first standardized perceived leader’s helping behavior and perceived leader’s role overload. Then we computed the interactive item of perceived leader’s helping behavior and perceived leader’s role overload. Model 6 in Table 6 shows that the interactive item was significantly related with voice behavior (*B* = 0.158, *SE* = 0.073, *p* < 0.05). Using the Excel sheets from Dawson’s website^1^, we visualized the moderating effect of perceived leader’s role overload as shown in Figure 2. Consistent with Aiken and West’s (1991) suggestion, we regarded the mean plus one standard deviation as a high value of perceived leader’s role overload and mean minus one standard deviation as a low value of perceived leader’s role overload. With R software, we conducted the parametric bootstrapping and ran a simple slope analysis. Consistent with Figure 2, the results showed that when perceived leader’s role overload was high, the effect of perceived leader’s helping behavior on voice behavior was significantly positive (*B* = 0.239, *SE* = 0.093, 95% CI for the indirect effect [0.060, 0.419]), while when perceived leader’s role overload was low, the positive effect of perceived leader’s helping behavior on voice behavior was not significant (*B* = 0.121, *SE* = 0.091, 95% CI for the indirect effect [−0.064, 0.302]). The effect of perceived leader’s helping behavior on voice behavior when perceived leader’s role overload is high is stronger than that when perceived leader’s role overload is low (difference = 0.119, *SE* = 0.057, 95% CI for the indirect effect [0.008, 0.227]). Hence, Hypothesis 3 was supported (Figure 2).

**FIGURE 3:**
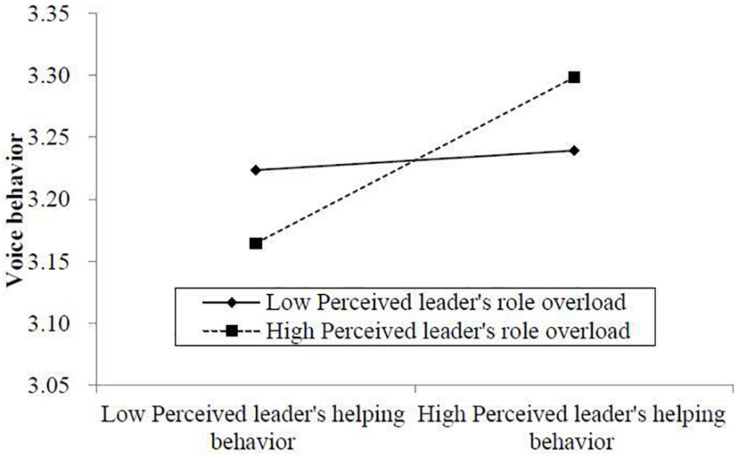
The moderating effect of perceived leader’s role overload on the indirect effect of perceived leader’s helping behavior via voice behavior.

As shown in Model 6 in Table 6, voice behavior has a positive effect on thriving at work (*B* = 0.186, *SE* = 0.055, *p* < 0.01). Combining the fact that perceived leader’s role overload moderates the relationship between perceived leader’s helping behavior and voice behavior, Hypothesis 4 was preliminarily supported. Then, we conducted the parametric bootstrapping and directly examined whether the indirect effect can be moderated by perceived leader’s role overload. Figure 3 shows that perceived leader’s role overload can moderate the indirect effect of voice behavior regarding the relationship between perceived leader’s helping behavior and voice behavior. The results showed that when perceived leader’s role overload is high the indirect effect of perceived leader’s helping behavior on thriving at work via voice behavior is significantly positive (Indirect = 0.045, *SE* = 0.022, 95% CI for the indirect effect [0.008, 0.090]), while when perceived leader’s role overload is low the positive effect of perceived leader’s helping behavior on thriving at work via voice behavior is not significant (Indirect = 0.022, *SE* = 0.019, 95% CI for the indirect effect [−0.010, 0.062]). Further, the mediating effect of voice behavior when perceived leader’s role overload is high is stronger than that when perceived leader’s role overload is low (Difference = 0.023, *SE* = 0.013, 95% CI for the indirect effect [0.001, 0.053]). Therefore, Hypothesis 4 was supported.

## Discussion

Using a diary research design, we demonstrate how leaders’ daily helping behavior promotes employees’ daily thriving. Specifically, we find that perceived leader’s helping behavior has a positive effect on employees’ thriving, and such an effect can be partially mediated by employees’ voice behavior. During such a mediating process, perceived leader’s role overload plays a role of accelerator, such that the positive association between perceived leader’s helping behavior and thriving at work is strengthened with the increase of perceived leader’s role overload. Besides that, perceived leader’s role overload can improve the mediating effect of voice behavior regarding the relationship between perceived leader’s helping behavior and thriving at work. These findings have significant implications for literature on thriving at work, leaders’ helping behavior, and social learning theory.

### Theoretical Implications

Accounting for thriving at work, we offer a new type of leadership behavior that fosters employees’ thriving. Previous studies have documented that authentic leadership (Xu et al., 2017), empowering leadership (Li et al., 2016), ethical leadership (Yousaf et al., 2019), service leadership (Jo et al., 2020), and many other leaders’ factors (e.g., Frazier and Tupper, 2018; Li et al., 2019; Yang et al., 2019) contribute to increasing employees’ thriving. However, all these studies accounted for between-person variance of thriving at work, and no research addressed the role of leaders’ helping behavior, which is a key part of a leadership role (Nadler et al., 2003). We examined the effect of perceived leader’s helping behavior on employees’ thriving and found that employees were able to keep thriving in their daily work when perceiving that their leaders assist them a lot in completing daily tasks. As previous studies suggested that thriving was a daily experience (Niessen et al., 2012; Prem et al., 2017), this study offered an answer about how employees’ daily thriving is cultivated by leaders’ daily helping behavior.

Further, this study provided a perspective of social learning theory to elaborate why employees’ thriving can be influenced by leaders’ behavior. According to social learning theory (Bandura, 1977), we posited that employees will learn the affiliative rule behind leaders’ helping behavior and engage in voice behavior, which is corresponding with the affiliative rule as well as helps them obtain leaders’ instructive feedback. With voice behavior, employees practice new learned rules and might correct their behavior though leaders’ feedback. During this process, employees experience thriving because of mastering new social skills and keeping competence. Our findings that voice behavior mediates the relationship between perceived leader’s helping behavior and employees’ thriving support the theoretical logic of social learning theory. However, the research that addresses the influence of leaders’ behavior on employees’ thriving has not considered social learning theory and regarded voice behavior as a kind of proactive learning behavior. The findings of this study can be also extended to other research associated with the relationship between leadership and employees’ thriving. For example, we can assume the mediating effect of voice behavior on the relationship between ethical leadership and employees’ thriving. Ethical leaders have high moral standards and point out inappropriate organizational activities publicly (Chen and Hou, 2016). Thus, employees will learn the rule that speaking out their concerns about organizational development is emphasized by leaders and engage in voice behavior. As indicated in our study, voice behavior is a means of imitating leaders’ behavior.

Accounting for leaders’ helping behavior, this study contributes to adding a new benefit of leaders’ helping behavior. Empirical studies have reported that leaders’ helping behavior is beneficial in increasing leader effectiveness (Asadullah et al., 2016) and relationship satisfaction (Hoption, 2016). Besides all these benefits, this study also indicates that employees will experience a sense of both learning and validity when they believe that their leaders offer assistance in completing tasks. Hence, this study enriches the literature about the outcomes of leaders’ helping behavior.

By examining the moderating role of perceived leader’s role overload, this study provides a boundary condition for the mediating effect of voice behavior on the relationship between perceived leader’s helping behavior and employees’ thriving. Specifically, we found that the indirect effect of perceived leader’s helping behavior on employees’ thriving via employees’ voice behavior was stronger when employees perceived high levels of leader’s role overload. Because of the logic that leaders should engage in the behavior that they deem as more rewarding when they experience role overload (Becker, 1965), employees have a high probability of concluding what kind of behavior is encouraged by leaders when acknowledging that leaders have to complete too much work. As social learning theory argues that people have a high tendency of learning the behavior that can produce valuable outcomes (Bandura, 1977), employees’ orientation of learning from their leaders is strong under the condition of perceived high levels of leader’s role overload. Hence, voice as a learning behavior is sensitively influenced by perceived leader’s helping behavior under such a condition. Furthermore, exploring the boundary condition of observational learning is an important question for social learning theory (e.g., Tu et al., 2018). Therefore, this finding might also contribute to social learning theory by pointing out that perceived leader’s role overload is a clue that motivates employees to observe and learn leaders’ behavior.

### Limitations and Future Study

Four limitations should be noted, with a view to future research. First, although we collected data at two different times in 1 day, which is helpful to address the casual effect, voice behavior and thriving at work were collected at the same time. Hence, it is difficult for us to clarify the causal relationship between voice behavior and thriving at work from our study. In fact, Yousaf et al. (2019) proposed a reversed relationship such that employees who are thriving at work are more likely to engage in voice behavior because thriving offers both new ideas and energy for employees to speak out. Despite this, we believe that our assumption that voice behavior as a kind of proactive learning behavior can increase employees’ thriving is theoretically sound. Indeed, future research is needed to better understand the causal relationship by adopting a cross-lagged design and measuring all variables at all-time points.

Second, all the variables were self-reported, which can lead to common method biases (Podsakoff et al., 2003). In order to control the impact of common method biases on our findings, we have collected data at two different times in 1 day. In addition, we also conducted a two-level CFA, and the hypothesized five-factor measurement model was significantly better than any other measurement model. This demonstrated that common method biases were not a serious concern in this study (Podsakoff et al., 2003). Despite this, future research can improve the research design by collecting data from different sources, such that we can collect leaders’ helping behavior and leaders’ role overload from the leader’s self-report, and voice behavior as well as thriving at work from employees.

Third, we have repeatedly measured perceived leader’s helping behavior, perceived leader’s role overload, felt obligation, voice behavior, and thriving at work for only 5 days. Although previous thriving literature that adopted a daily diary design all collected 5-day data (Niessen et al., 2012; Prem et al., 2017), the small timeframe might be not enough to capture the micro-processes within persons. Hence, future research should reexamine our hypothesis by collecting data in a larger timeframe (e.g., 2 weeks).

Finally, the sample size of this study was small, and all participants were from China. These two drawbacks might constrain the universality of our findings. It is better to collect more data and examine the moderated mediating model in other cultural contexts. However, examining this model in the Chinese context might have its own advantage. As the Chinese culture tends to lean more toward collectivism (Fan, 2000), people are more likely to take cooperative behavior for granted because it is consistent with social culture. This collective culture makes it easier to underestimate the value of helping behavior and invalidate the moderated mediating model. Hence, the significant findings in our study might be more established in other non-collectivist cultures.

### Practical Implications

The findings of our study have important practical implications for organizations to maintain thriving at work. First, it is advocated that leaders should offer employees assistance in tasks because the findings of this study show that employees who receive leaders’ help are more likely to feel a sense of learning and validity. Second, leaders should be aware that their work behavior has a large effect on employees’ voice behavior, especially when they suffer from role overload. According to our findings that employees tend to imitate leaders’ helping behavior, using voice behavior, and acquire the experience of thriving when acknowledging that leaders come across as having role overload, leaders should engage in helping behavior more frequently despite experiencing role overload. As an extension of this finding, leaders who have high levels of role overload should attempt to avoid negative work behavior (e.g., counterproductive work behavior), which has a high probability of causing employees to learn these negative work behaviors, because employees’ orientation of learning from leader behavior might be high under this condition. Third, considering that voice behavior has a positive effect on employees’ thriving, encouraging employees to express their ideas is also a useful approach. According to previous findings, various ways can be applied to increase voice behavior, such as reducing job stressors (Ng and Feldman, 2012), developing high-quality supervisor–subordinate *guanxi* (Wang et al., 2019), enhancing employees’ organization-based self-esteem (Liang et al., 2012), and so on (e.g., Liu et al., 2010; Grant, 2013; Zhang et al., 2015).

## Conclusion

In conclusion, our study found a positive effect of perceived leader’s helping behavior on employees’ thriving and that employees’ voice behavior can partially mediate such impact. Moreover, the association between perceived leader’s helping behavior and employees’ thriving as well as the mediating effect of employees’ voice behavior can be strengthened by perceived leader’s role overload. Our study contributes to illustrating how and when employees’ daily thriving is shaped by leaders’ daily helping behavior.

## Data Availability Statement

The raw data supporting the conclusions of this article will be made available by the authors, without undue reservation, to any qualified researcher.

## Ethics Statement

Ethical review and approval was not required for the study on human participants in accordance with the local legislation and institutional requirements. Written informed consent for participation was not required for this study in accordance with the national legislation and the institutional requirements.

## Author Contributions

LC and Z-DZ drafted and designed the work. Z-DZ and W-TJ collected the data and critically revised the manuscript. LC analyzed the data and drafted the manuscript. All authors gave final approval of the manuscript before submission.

## Conflict of Interest

The authors declare that the research was conducted in the absence of any commercial or financial relationships that could be construed as a potential conflict of interest.

## Footnotes

1 http://www.jeremydawson.com/slopes.htm
